# Real-time observation of frequency Bloch oscillations with fibre loop modulation

**DOI:** 10.1038/s41377-021-00494-w

**Published:** 2021-03-05

**Authors:** Hao Chen, NingNing Yang, Chengzhi Qin, Wenwan Li, Bing Wang, Tianwen Han, Chi Zhang, Weiwei Liu, Kai Wang, Hua Long, Xinliang Zhang, Peixiang Lu

**Affiliations:** School of Physics and Wuhan National Laboratory for Optoelectronics, Huazhong University of Science and Technology, Wuhan, 430074 China

**Keywords:** Fibre optics and optical communications, Photonic devices, Frequency combs

## Abstract

Bloch oscillations (BOs) were initially predicted for electrons in a solid lattice to which a static electric field is applied. The observation of BOs in solids remains challenging due to the collision scattering and barrier tunnelling of electrons. Nevertheless, analogies of electron BOs for photons, acoustic phonons and cold atoms have been experimentally demonstrated in various lattice systems. Recently, BOs in the frequency dimension have been proposed and studied by using an optical micro-resonator, which provides a unique approach to controlling the light frequency. However, the finite resonator lifetime and intrinsic loss hinder the effect from being observed practically. Here, we experimentally demonstrate BOs in a synthetic frequency lattice by employing a fibre-loop circuit with detuned phase modulation. We show that a detuning between the modulation period and the fibre-loop roundtrip time acts as an effective vector potential and hence a constant effective force that can yield BOs in the modulation-induced frequency lattices. With a dispersive Fourier transformation, the pulse spectrum can be mapped into the time dimension, and its transient evolution can be precisely measured. This study offers a promising approach to realising BOs in synthetic dimensions and may find applications in frequency manipulations in optical fibre communication systems.

## Introduction

Bloch oscillations (BOs) describe the periodic movement of electrons in solids to which an external static electric field is applied^1,2^ and are associated with a variety of interesting phenomena concerning the ultrafast transport of electrons. Observation of BOs in real time reveals the physical origins of the phenomena. However, it is very challenging to measure BOs directly in natural solids since the relaxation time of electrons is usually much shorter than the oscillation period. To date, analogies of electron BOs for neutral particles such as photons, acoustic phonons and cold atoms have been demonstrated in waveguide arrays^3–5^, acoustic cavities^6,7^ and optical lattices^8,9^, which offer alternative platforms to investigate BOs more conveniently.

Optical BOs in waveguide arrays stem from discrete diffraction^10–14^. This concept has been extended to synthetic dimensions of time^15,16^, frequency^17–19^ and angular momenta^20,21^. Recently, much research interest has been devoted to BOs in synthetic frequency lattices, which are usually formed by imposing temporal modulation on dielectric waveguides or fibres^22–24^. Modes with periodic frequencies in the lattice can couple to each other and experience discrete frequency diffraction. With the introduction of a temporal walk-off between the modulating field and the incident optical pulse, frequency BOs have been experimentally demonstrated in a nonlinear fibre with cross-phase modulation^23^. The effective electric-field force arises owing to a scalar potential formed by the temporal walk-off. However, the frequency spectrum has been obtained only at the output of the fibre, and the evolution process of BOs has been measured only indirectly. In addition, frequency BOs have been theoretically demonstrated in micro-resonators under temporal modulation^24^, in which the frequency lattice is constructed based on the resonating modes. The driving force originates from the vector potential induced by a detuning between the modulation frequency and the modal frequency spacing. The optical vector potential has currently attracted intensive attention in topological photonics for its unique role in manipulating photons^25^. Considering the compact structure of ring resonators, the direct observation of BOs still faces difficulties in compensating for the power reduction when collecting signals. Moreover, as a transient process, transient oscillation is difficult to observe directly by using traditional spectroscopy techniques.

In this contribution, we perform an experimental study of frequency BOs in a fibre loop including an optical phase modulator. The phase modulation produces a frequency lattice in which optical modes with discrete frequencies can couple to each other, resulting in discrete frequency diffraction. A small detuning is introduced between the modulation period and pulse circulation time in the fibre loop, which serves as an effective electric-field force in the frequency lattice and thus gives rise to frequency BOs^26,27^. We show that the vector potential can also contribute to the generation of the effective force, which varies with the propagation distance. The frequency evolution is measured by a home-made spectroscopy device based on the dispersive Fourier transformation (DFT)^28–31^. Hence, the rapidly changed spectrum can be precisely recorded in real time. By means of BOs, the frequency spectrum in the telecommunication band can be shifted by as much as hundreds of GHz. The spectrum for the incidence of the narrowband experiences a breathing motion within a wide range of 300 GHz. The results may find many applications in signal processing and frequency comb manipulation^32–34^. Since the period and amplitude of BOs are sensitive to the optical length of the fibre loop, this study may also enable precise fibre sensing^35–37^.

## Results

### Theoretical model of frequency BOs in fibre loops under time-detuned modulation

**Fig. 1:**
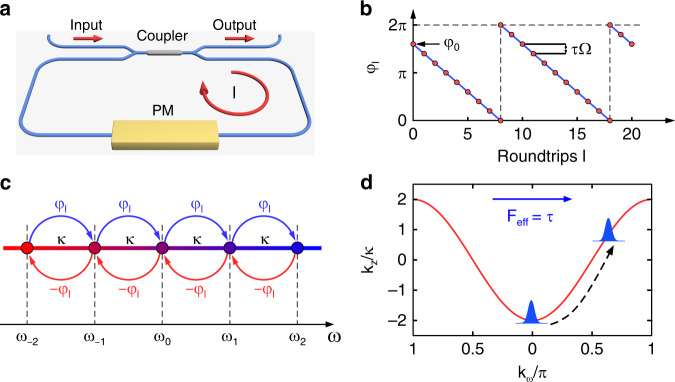
Conceptual diagram of the artificial frequency lattice and effective force in the phase modulator. **a** Schematic sketch of the fibre loop. *l* denotes the number of roundtrips. The red arrows indicate the propagation direction of the pulse. **b** Schematic diagram of the phase *φ*_*l*_. The red dots denote the phase of the modulation signal. *τ* is the time detuning between the optical pulse and modulation signal. Ω and *φ*_0_ are the frequency and initial phase of the modulation signal, respectively. **c** Mode coupling in the vicinity of *ω*_0_. *κ* is the coupling strength between the adjacent modes. *φ*_*l*_ and −*φ*_*l*_ denote the phase shifts for upward and downward transitions in the frequency. **d** Dispersion relation of the frequency Bloch mode for *φ*_*l*_ = 0. The time detuning *τ* plays the role of effective electric-field force *F*_eff_

We consider an optical fibre loop that includes an optical phase modulator (PM), as shown in Fig. 1a. The incident optical pulse can couple into and out of the fibre loop through a 50/50 directional coupler. The PM undergoes a travelling-wave modulation of the refractive index as *n*_d_(*t*, *z*) *= n*_0_ + Δ*n*cos(Ω*t − qz* + *φ*_0_), where *n*_0_ is the background refractive index, *z* is the local coordinate along with the PM and Δ*n* denotes the modulation amplitude. Ω, *q* and *φ*_0_ represent the frequency, wavevector and initial phase of the modulation, respectively. The modulation can induce photonic transitions among a discretized set of optical modes with frequency *ω*_*n*_ = *ω*_0_ + *n*Ω (*n* = ±1, ±2, …), forming a discrete frequency lattice with period Ω. The neighbouring modes with frequency spacing Ω experience upward and downward transitions between each other. The propagation constants of the modes are given by *β*_*n*_ = *β*_0_ + *nq*, with *q* = Ω/*v* and *v* being the light speed in the PM. Considering that Ω ≪ *ω*_0_, the group velocity dispersion (GVD) can be neglected, and the dispersion relation is approximately linear. Consequently, the wavevector matching condition is automatically satisfied.

We start by considering the resonant modulation when the roundtrip time *T*_*L*_ equals integer multiples of the modulation period *T*_*M*_ = 2*π*/Ω. The frequency transitions in the PM can be described by the following tight-binding Hamiltonian:

1 $$H = \kappa \mathop {\sum}\limits_n {\left[ {e^{i\varphi _l}\left| {n + 1} \right\rangle \left\langle n \right| + e^{ - i\varphi _l}\left| {n - 1} \right\rangle \left\langle n \right|} \right]}$$

We denote the eigenstate in the frequency lattices as |Ψ(*z*)〉 = ∑_*n*_*a*_*n*_(*z*)|*n*〉, where *a*_*n*_(*z*) is the complex amplitude of mode |*n*〉 at frequency *ω*_*n*_. By substituting the eigenstate into the Schrodinger equation *i∂*_*z*_ |Ψ(*z*)〉 = *H* |Ψ(*z*)〉, we can obtain the coupled-mode equation (see Supplementary Information for the detailed derivation)

2 $$i\frac{{\partial a_n\left( z \right)}}{{\partial z}} = \kappa \left[ {e^{i\varphi _l}a_{n - 1}\left( z \right) + e^{ - i\varphi _l}a_{n + 1}\left( z \right)} \right]$$

where *κ* = Δ*nk*_0_/2 denotes the coupling coefficient between adjacent frequency modes, with *k*_0_ being the vacuum wavevector. *φ*_*l*_ refers to the photonic analogue of the Peierls phase accompanying the upward frequency transition. For the downward transition, the phase becomes −*φ*_*l*_^38,39^. The phase factor corresponds to an effective gauge potential applied along the propagation direction of the frequency lattice^17,40^. In this case, the modulation phase and the associated gauge potential remain constant, i.e. *φ*_*l*_ = *φ*_0_. Hence, no effective electric field exists, and BOs cannot occur.

Analogous to Bloch modes in the spatial lattice, the Bloch mode in the frequency lattice refers to an ideal frequency comb possessing infinite width and uniform amplitude, which has the form *a*_*n*_ = *a*_0_exp(*ink*_*ω*_Ω)exp(*ik*_*z*_*z*), where *k*_*ω*_ is the Bloch wavevector and *k*_*z*_ is the collective propagation constant along the *z* direction. According to Eq. (2), the dispersion relation for the Bloch mode in the frequency lattice is given by *k*_*z*_ = −2*κ*cos(*k*_*ω*_Ω − *φ*_0_).

When a time detuning *τ* is introduced between *T*_*L*_ and *T*_*M*_ in each roundtrip, defined as *τ* = mod(*T*_*L*_, *T*_*M*_), the dispersion relation becomes *k*_*z*,*l*_ = –2*κ*cos(*k*_*ω*_Ω – *φ*_*l*_), with *φ*_*l*_ = *φ*_0_ – *l*Ω*τ* being the modulation phase in the *l*-th roundtrip. As shown in Fig. 1b, the presence of time detuning produces a linear variation in the modulation phase. The dispersion relation can be rewritten as

3 $$k_{z,l} = - 2\kappa {\mathrm{cos}}\left[ {\left( {k_\omega + l\tau } \right){\Omega} - \varphi _0} \right]$$

which implies that the linear variation in the modulation phase is equivalent to a shift of the Bloch wavevector. Similar to electrons moving in the electromagnetic field, the shift of the Bloch wavevector here can be attributed to the presence of an effective vector potential *A*_eff_(*l*) = −*lτ*, which varies linearly with the roundtrip numbers and corresponds to a constant effective electric-field force *F*_eff_ = −d*A*_eff_/d*l* = *τ*. The force is responsible for BOs in the frequency lattice^22,41^.

Considering the incidence of a finite-width frequency comb, the effective group velocity in the frequency dimension is *v*_*g*,*l*_ = −∂*k*_*z*,*l*_/*∂k*_*ω*_ = −2*κ*Ωsin[(*k*_*ω*_ + *lτ*)Ω − *φ*_0_]. Thus, the spectrum shift in the *l*-th roundtrip is Δ*ω*(*l*) = *v*_*g*,*l*_*L* = −*m*_*φ*_Ωsin[(*k*_*ω*_ + *lτ*)Ω – *φ*_0_], where *m*_*φ*_ = 2*κL* is the phase modulation depth and *L* is the length of the PM. As the roundtrip number increases, the accumulated frequency shift becomes $\omega = \omega _0 + \mathop {\int}\limits_0^l {{\Delta}{\upomega}\left( {l^{\prime}} \right){\mathrm{d}}l^{\prime}}$. Hence, the centre frequency follows a motion governed by

4 $$\omega = \omega _0 + \frac{{m_\varphi }}{\tau }\left\{ {\left. {{\mathrm{cos}}\left[ {\left( {k_\omega + l\tau } \right){\Omega} - \varphi _0} \right] - {\mathrm{cos}}\left( {k_\omega {\Omega} - \varphi _0} \right)} \right]} \right\}$$

where *ω*_0_ stands for the initial input centre frequency. The equation suggests that the centre frequency experiences a periodic oscillation with the number of roundtrips, referring to the frequency BOs. The oscillation period and amplitude of the BOs are given by *Z*_BO_ = 2*π*/|*τ*Ω| and *A*_BO_ = *m*_*φ*_/|*τ*|, respectively. Both values are inversely proportional to the time detuning *τ*. In addition, the initial phase of the BOs is determined by the phase difference Δ*φ* = *k*_*ω*_Ω − *φ*_0_.

On the other hand, for a broad pulse input with a very narrow frequency spectrum, it can be regarded as a continuous wave containing only a single frequency, with the initial amplitude given by *a*_*n*_(0) = *a*_0_*δ*_*n*,0_. By combining this equation with Eq. (2), we can obtain the spectrum evolution^13^

5 $$\left| {a_n\left( l \right)} \right| = a_0\left| {J_n\left[ {\frac{{2m_\varphi }}{{\tau {\Omega}}}\sin \left( {\frac{{\tau {\Omega}}}{2}l} \right)} \right]} \right|$$

which also coincides with the oscillation period *Z*_BO_ = 2π/|*τ*Ω|. Thus, the spectrum manifests a breathing pattern accompanied by a self-focusing effect during evolution. In the following experiments, we implement the incidence of both short and broad pulses and directly observe the oscillating and breathing modes of frequency BOs.

### Experimental realisation of frequency BOs in a modulated fibre loop

**Fig. 2:**
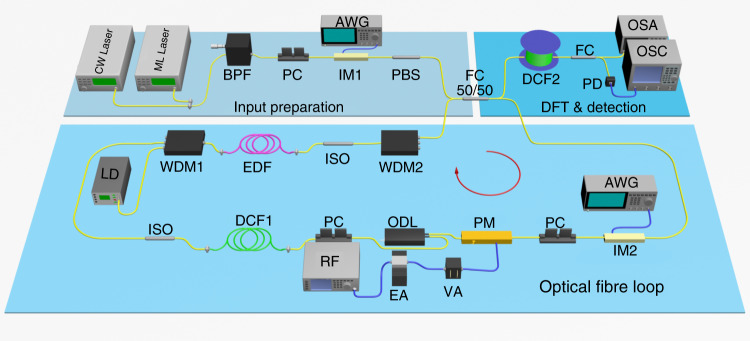
Schematic of the experimental setup based on a fibre-loop circuit. CW Laser continuous-wave laser, ML Laser mode-locked laser, BPF bandpass filter, PC polarisation controller, AWG arbitrary waveform generation, IM intensity modulator, PBS polarisation beam splitter, FC fibre coupler, RF radio-frequency signal generator, EA electrical amplifier, VA variable attenuator, ODL optical delay line, DCF dispersion-compensating fibre, ISO isolator, LD laser diode, WDM wavelength-division multiplexer, EDF erbium-doped fibre, PD photodetector, OSC oscilloscope, OSA optical spectrum analyser. The red circle represents the propagation direction of the circling pulse

The experimental setup for demonstrating frequency BOs is shown in Fig. 2. An optical pulse with a width *T*_0_ is injected into the fibre loop through a 50/50 coupler. The PM is driven by a cosinoidal radio frequency (RF) signal with a frequency Ω/2*π* = 10 GHz. An electrical amplifier (EA) and variable attenuator (VA) are employed to change the applied voltage on the PM and adjust the modulation depth *m*_*φ*_. The optical delay line (ODL) is utilised to adjust the loop roundtrip time *T*_*L*_ and introduce the time detuning *τ*. Then, the modulation signal experiences a time shift proportional to the number of roundtrips with respect to the incident pulse. To efficiently characterise the frequency BOs, the time detuning should be large enough to guarantee considerable oscillation periods within a few roundtrips before the loss and noise dominate. On the other hand, it has to be small enough to maintain a significant oscillation amplitude. The dispersion-compensating fibre (DCF1) compensates for the anomalous fibre dispersion in the fibre loop. Thus, we consider only the influence of the PM on the pulse spectrum. The number of roundtrips *l* is controlled by the intensity modulator (IM2). To obtain a higher signal-to-noise ratio, the propagation and insertion loss of the fibre loop is only partially compensated by the erbium-doped fibre (EDF), which is pumped by a laser diode (LD). The energy coupled out from the loop after each roundtrip is also compensated by the EDF.

To realise real-time measurement of the pulse spectrum coupled out from the coupler, a spectroscope based on the DFT is connected at the end of the fibre-loop circuit. The pulse first enters a 16-km-long DCF2, the GVD of which is given by *β*_2_ = 210 ps^2^/km. The objective of DCF2 is to perform a Fourier transform, which maps the spectrum envelope of the optical pulse into a time-domain waveform^28,29^. Thanks to the dispersion in DCF2, real-time measurement of the frequency spectrum with a resolution of ~9.8 GHz can be achieved. When a 40-GHz-bandwidth photodetector (PD) is employed, the temporal waveform is converted to electric signals and recorded by a high-speed real-time oscilloscope (OSC). The bandwidth and sampling rate of the oscilloscope are 33 GHz and 80 GSa/s, respectively. At the same time, the time-averaged spectrum is detected by an optical spectrum analyser (OSA). The averaged spectrum is employed to verify the frequency-to-time mapping in the DFT technique.

**Fig. 3:**
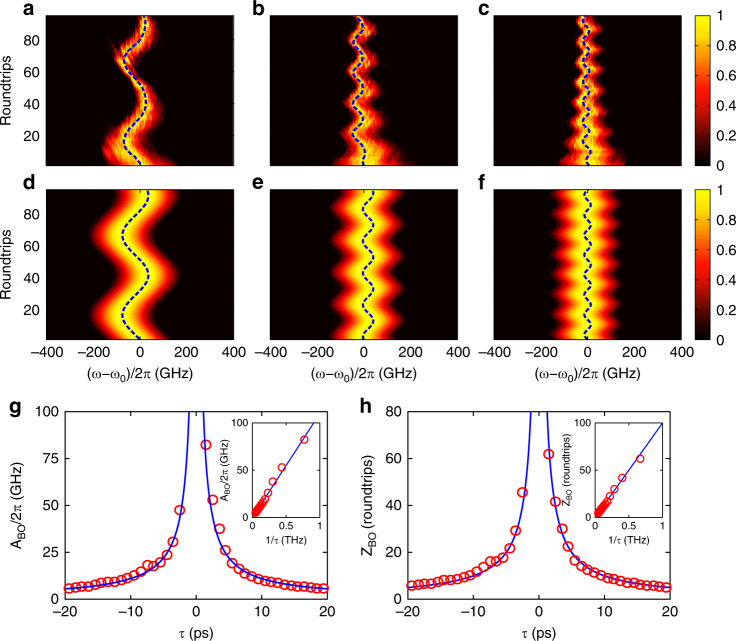
BOs in the frequency dimension for *T*_0_*=* 3.5 ps. **a**–**c** Experimental results of the frequency BOs under a time detuning of *τ* = 2, 5 and 8 ps. Here, we set *m*_*φ*_ *=* 0.7. **d**–**f** Simulated BOs corresponding to the experimental results in (**a**–**c**). The blue dashed lines denote the trajectories of the centre frequency. **g**, **h** Amplitude and period of the BOs as a function of the time detuning *τ*. The red circles and blue lines represent the experimental and theoretical results, respectively. The insets show the relations for *A*_BO_ and *Z*_BO_ versus 1/|*τ*|

In the fibre loop, the PM is driven by the periodic RF signal, which creates a frequency lattice with a period of Ω/2*π* = 10 GHz. The modulation depth is *m*_*φ*_ = 0.7. The reciprocal vector of the frequency lattice *k*_*ω*_ takes values in the range [–π/Ω, π/Ω], which refers to the first Brillion zone. The spectrum of the incident pulse can be regarded as the superposition of a series of frequency Bloch modes with different values of *k*_*ω*_. The range of *k*_*ω*_ is determined by the width of the incident pulse^42^. Figure 3 shows the spectrum evolution of the short pulse with width *T*_0_ = 3.5 ps. Although the pulse spectrum is continuous, it can be treated as the superposition of a series of frequency combs propagating in corresponding individual frequency lattices. The BOs in each lattice share the same features, except for a lateral frequency shift. The theoretical analysis is still valid for the incidence of a continuous spectrum. Because the pulse width is much smaller than the modulation period 2*π*/Ω = 100 ps, the spectrum envelope of the short pulse has a larger uncertainty in the reciprocal vector *k*_*ω*_ and undergoes less frequency diffraction. When the length of the ODL is adjusted, the time detuning *τ* becomes tuneable. The evolution patterns of frequency BOs for *τ* = 2, 5 and 8 ps are shown in Fig. 3a–c, respectively. The trajectories of the centre frequency are plotted as the blue dashed curves. One sees that the spectrum of the incident pulse evolves along a cosinoidal trajectory as the number of roundtrips increases. The measured periods of the BOs are *Z*_BO_ ≈ 50, 20 and 13 roundtrips, which agree well with the theoretical values obtained by *Z*_BO_ = 2*π*/|*τ*Ω|. The corresponding oscillation amplitudes of the BOs are *A*_BO_/2*π* ≈ 48, 25 and 14 GHz. It should be mentioned that the initial phase of the BOs is uncertain since the incident pulse undergoes a time drift with respect to the modulation signal. Every time the pulse is coupled into the fibre loop, the time drift takes different values. The initial phases of the BOs can be determined from the trajectories of the centre frequency in Fig. 3a–c, which are Δ*φ* ≈ 0.4*π*, 0.7*π* and 0.4*π*. We also numerically simulate the BOs for different values of time detuning by using the modified split-step Fourier algorithm^43,44^, as shown in Fig. 3d–f. The initial phases are extracted from the experiments. The evolution patterns coincide fairly well with the experimental data, except for the spectral widths. Because the gain spectrum of the EDF is not uniform in practice, different frequency components of the pulse suffer distinct amounts of loss. As the number of roundtrips increases, the amplitude of the spectrum decreases nonlinearly in different frequency regions, resulting in the narrowing of the spectral width. More details concerning the influence of the gain spectrum are provided in the Supplementary Information. To obtain a high signal-to-noise ratio experimentally, the loss is only partially compensated for since the EDF amplifies both the signal and noise. As the pump power increases, the noise level rises faster than the signal and may drown the effect of the BOs.

The oscillation amplitude and period as functions of the time detuning are shown in Fig. 3g, h, respectively. The red circles denote the experimental data. According to Eq. (4), both the amplitude and period are inversely proportional to the absolute value of the time detuning. The analytical results are depicted by the blue curves. Changing the sign of *τ* leads to a mirror symmetry of the oscillation trajectory. The time detuning *τ* results in an increase in the Bloch wavevector for *τ* > 0 and a decrease for *τ* < 0. When *τ* = 0, the spectrum exhibits directional transport, with the diffraction being suppressed at Δ*φ* = −*π*/2 or *π*/2. The experimental results of *A*_BO_ and *Z*_BO_ as functions of 1/|*τ*| are depicted by the red circles in the insets of Fig. 3g, h. The values of *A*_BO_ and *Z*_BO_ vary linearly with 1/|*τ*|. The linear relationship agrees closely with the theoretical predictions by Eq. (4).

**Fig. 4:**
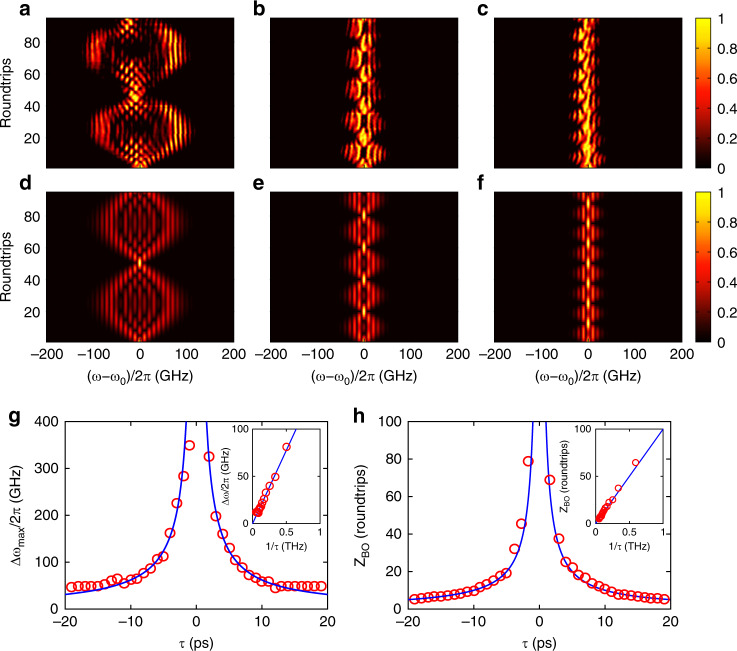
Breathing patterns of the frequency BOs for the pulse duration *T*_0_ = 140 ps. **a**–**c** Experimental spectrum evolution with *τ =* 2, 5 and 8 ps, respectively. The modulation depth is *m*_*φ*_ *=* 0.45. **d**–**f** Numerical results corresponding to (**a**–**c**). **g**, **h** Dependence of the oscillation amplitude *A*_BO_ and oscillation period *Z*_BO_ on the time detuning *τ*. The red circles and blue lines represent the experimental and theoretical results, respectively. The insets in **g** and **h** show the experimental data of *A*_BO_ and *Z*_BO_ versus 1/|*τ*|

For a broader pulse with *T*_0_ = 140 ps, the initial spectrum should be remarkably narrowed. As the number of roundtrips increases, the spectrum gradually becomes wider, similar to discrete diffraction in the frequency lattice^10,17^. The narrowed spectrum can be regarded as a superposition of many Bloch modes with wavevectors *k*_*ω*_ covering the whole Brillouin zone, which undergo BOs with different amplitudes. Because of the time detuning, the BOs for distinct Bloch modes have identical oscillation periods. As a result, the spectrum envelope manifests a breathing pattern, as shown in Fig. 4. For *m*_*φ*_ = 0.45, the experimental results of the frequency BOs are as illustrated in Fig. 4a–c for *τ* = 2, 5 and 8 ps. The oscillation periods are ~50, 20 and 13 roundtrips. The values of the corresponding maximum spectral broadening are Δ*ω*_max_/2*π* = *A*_BO_/*π* ≈ 226, 83 and 62 GHz, respectively. Compared with the numerical results shown in Fig. 4d–f, the value of Δ*ω*_max_ measured in the experiment gradually decreases during evolution. The deviation originates from the non-flat gain spectrum of the EDF, which results in different attenuations for distinct frequency components. In addition, the asymmetric breathing patterns shown in Fig. 4a–c are caused by fibre dispersion in the loop. As the number of roundtrips increases, the dispersion effect accumulates and induces remarkable time delays for individual frequency components. The details can be found in the Supplementary Information. The influence of time detuning on the maximum spectral broadening Δ*ω*_max_ and oscillation period *Z*_BO_ is illustrated in Fig. 4g, h. Meanwhile, Δ*ω*_max_ and *Z*_BO_ versus 1/|*τ*| are shown in the insets. The red circles denote the experimental data, which agree with the analytical results plotted by the blue curves. When *τ* = 0, the effective electric-field force *F*_eff_ vanishes, and the spectrum evolution corresponds to normal discrete diffraction^10,11^. Then, Δ*ω*_max_ increases linearly with the number of roundtrips, and a wider frequency comb can be generated.

**Fig. 5:**
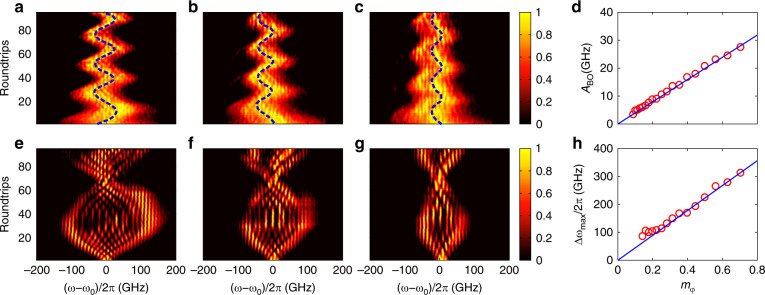
Frequency BOs for different electrical attenuations. **a**–**c** Spectrum evolutions of the frequency BOs for electrical attenuations equal to 0, 3 and 6 dB when *T*_0_ = 3.5 ps and *τ* = 4 ps. The blue curves denote the trajectories of the centre frequency. **d** Oscillation amplitude *A*_BO_ as a function of the modulation depth *m*_*φ*_. The red circles and blue line denote the experimental and theoretical results, respectively. **e**–**g** Breathing patterns of the frequency BOs corresponding to **a**–**c** when *T*_0_ = 140 ps and *τ* = 1.5 ps. **h** Maximum spectral broadening Δ*ω*_max_ versus *m*_*φ*_ when *T*_0_ = 140 ps and *τ* = 1.5 ps

The oscillation amplitude of BOs can also be controlled by the modulation depth, which determines the coupling strength between the adjacent frequency modes and can be attenuated by using an EA. For *T*_0_ = 3.5 ps and *τ =* 4 ps, the spectrum evolution manifests a shift along a cosinoidal trajectory with a period of *Z*_BO_ = 25 roundtrips, as shown in Fig. 5a–c. The initial modulation depth is *m*_*φ*_ *=* 0.7. Because the attenuations selected are 0, 3 and 6 dB, the oscillation amplitude of the frequency BOs can be switched to *A*_BO_/2*π* ≈ 28, 21 and 14 GHz. The initial phases of the BOs are Δ*φ* ≈ –0.3*π*, –0.4*π* and 0.3*π*. The linear relationship between *A*_BO_ and *m*_*φ*_ is confirmed by sweeping the attenuation from 0 to 20 dB, as shown in Fig. 5d. The experimental data are indicated by the red circles and coincide well with the theoretical results in Eq. (4). A larger frequency shift based on BOs can be realised by increasing the amplitude of the modulation depth.

Next, we change the pulse width and time detuning to *T*_0_ = 140 ps and *τ* = 1.5 ps, respectively. The spectrum evolution manifests a breathing pattern with a period of 66 roundtrips. The modulation depth is set to *m*_*φ*_ *=* 0.7. As the attenuation changes from 0 to 3 and 6 dB, the spectra are broadened up to Δ*ω*_max_/2*π* ≈ 312, 226 and 168 GHz, as shown in Fig. 5e–g. The linear relationship between Δ*ω*_max_ and *m*_*φ*_ is confirmed in Fig. 5h. The experimental data and analytical results are denoted by the red circles and blue lines, respectively. The results show that the method is flexible enough to manipulate the spectrum by changing the modulation depth. The spectrum extension achieved in our system can be extended to hundreds of GHz, which improves the efficiency of electric-optical modulation.

## Discussion

We directly observed the frequency BOs in a fibre loop under phase modulation with time detuning. The spectrum of the incident optical pulse experiences a periodic oscillation in the frequency lattice formed by the phase modulation. The time detuning produces an effective electric-field force in the lattice, which is associated with the effective vector potential varying with the spectrum evolution. Additionally, the transient evolution of the spectrum was measured in real time. Based on the frequency-domain BOs, a maximum frequency shift up to 82 GHz was achieved. The bandwidth of the input pulse was also broadened up to 312 GHz. The spectrum manipulations overcome the microelectronic bandwidth limitation. This study may find many applications in high-efficiency frequency conversion and signal processing.

For discrete systems, the dispersion relation along the synthetic dimension plays a critical role in predicting the mode evolution. Thus, the effect of BOs can be employed to reveal the intrinsic properties of the synthetic dimension^42^. By changing the modulation signal or cascading multiple modulators, our system opens the possibility for experimental studies of other electron effects in the frequency dimension, such as super-Bloch oscillations^45^ and dynamic localisation^46,47^. Motivated by a recent work on long-range coupling^48,49^, frequency BOs may be realised in high-dimensional frequency crystals by driving the PM with multiple RF signals in the fibre loop. The effect of frequency BOs can also be extended from classical to quantum light, which may be used for the frequency manipulation of single-photon sources^50,51^. An optical fibre loop incorporated with a PM can be employed to simulate photonic time crystals. The system also enables the investigation of solitons^44^, parity-time symmetry^52,53^ and edge states^54^ in the synthetic time dimension.

## Materials and methods

### Details of the experimental setup

The experimental setup is shown in Fig. 2. In the experiment, the cavity length was ~28.5 m, corresponding to a roundtrip frequency of ~7 MHz. To ensure that only one pulse propagates in 100 roundtrips without another pulse entering the loop, the repetition rate of the input should be less than 7 MHz/100 = 70 kHz. The two optical sources were utilised separately to generate optical pulses with different widths. A mode-locked (ML) laser was employed to generate a pulse train. The centre wavelength, repetitive frequency and pulse width were 1555 nm, 19.93 MHz and 3.5 ps, respectively. To reduce the repetition rate of the input ML laser, a synchronised programmable signal with a frequency of 19.93 MHz/300 = 66.43 kHz was generated by using an arbitrary waveform generator. The signal drove IM1 to open a 30 ns time window. Then, the repetitive frequency of the pulse train was switched to 66.43 kHz. In addition, with the use of a continuous-wave (CW) laser, single-frequency light with a wavelength of 1555 nm was generated. When the duty ratio of the programmable signal was altered, the continuous wave was truncated by IM1 and transformed into an input pulse with a width of 140 ps. Another programmable signal with an adverse duty ratio was also generated and applied to IM2. Thus, the roundtrip number of the circling pulse was controllable. The noise of the input was filtered by an 8-nm band-pass filter (BPF). To excite only one polarisation mode matching the principal axis of the modulators, the polarisation state of the pulse could be controlled by using a polarisation beam splitter (PBS) and polarisation controller (PC). The 3.8-m-long DCF1 was used to compensate for the anomalous dispersion in the fibre loop. Unidirectional propagation was ensured by using isolators (ISOs). The 7.5-m-long EDF was pumped by a 980 nm laser diode (LD). The provided gain was 17 dB. Then, the pump laser could be filtered out by using the wavelength-division multiplexer (WDM).

### Mapping relationship between the spectrum and temporal waveform

To achieve real-time measurement of the frequency spectrum of the pulse coupled out from the coupler, the DFT technique is adopted at the end of the circuit. The spectrum envelope can be mapped into the temporal waveform of the pulse through fibre dispersion^28,29^. The mapping relationship between frequency and time is given by ${\Delta}f = \left[ {c{\Delta}t{\mathrm{/}}\left( {\lambda _0^2\left| {{\mathrm{DL}}_D} \right|} \right)} \right]$, where Δ*f* and Δ*t* are the precision of the frequency and time, respectively. *L*_*D*_ is the length of the fibre, and $D = -2\pi c\beta _2/\lambda _0^2$ denotes the dispersion coefficient, with *λ*_0_ being the centre wavelength. In the experiment, *L*_*D*_ is ∼16 km, and *λ*_0_ is 1555 nm. The width of the spectrum could be calculated according to the mapping relationship and the measured temporal waveform. Furthermore, the shift in the centre frequency leads to a varying time interval between the recorded pulses. Thus, the frequency shift can be obtained by taking the difference between the time interval and roundtrip time.

## Supplementary Information

Supplementary Information for: Real-time observation of frequency Bloch oscillations with fibre loop modulation
