# Supplementary material for: Real-time observation of frequency Bloch oscillations with fibre loop modulation
Source: Light Sci Appl. 2021 Mar 5;10:48. doi: 10.1038/s41377-021-00494-w (PMC7935930; doi:10.1038/s41377-021-00494-w)
Supplement: Supplementary file 1 — Supplementary Information for: Real-time observation of frequency Bloch oscillations with fibre loop modulation [file 41377_2021_494_MOESM1_ESM.docx]

**Supplementary Information for:**

**Real-time observation of frequency Bloch oscillations with fibre loop modulation**

Hao Chen^†1^, NingNing Yang^†1^, Chengzhi Qin^†1^, Wenwan Li^1^, Bing Wang^1^ ⃰, Tianwen Han^1^, Chi Zhang^1^ ⃰, Weiwei Liu^1^, Kai Wang^1^, Hua Long^1^, Xinliang Zhang^1^ and Peixiang Lu^1^ ⃰

^1^School of Physics and Wuhan National Laboratory for Optoelectronics, Huazhong University of Science and Technology, Wuhan, 430074, China

⃰ E-mails:

[wangbing@hust.edu.cn](mailto:wangbing@hust.edu.cn);

chizheung@hust.edu.cn;

lupeixiang@hust.edu.cn

^†^These authors contributed equally to this work.

**I. coupled-mode equation in the frequency lattice**

Evolutions of the spectrum occurring in LiNbO_3_ electro-optic phase modulator (PM) can be analysed from the perspective of travelling-wave modulation. In the PM, the crystal refraction index is driven by a cosine radiofrequency (RF) signal. We assume that the modulation is uniform in the transverse direction. Thus the time-varying refraction index is given by­­­­­­

, (S1)

where *n*_0_ is the background refractive index, with Δ*n*, Ω, *q*, and *φ*_0_ being the modulation amplitude, frequency, wavevector and initial phase, respectively. The instantaneous dielectric modulation of the waveguide is *ε*(*z*, *t*) = *n*(*z*, *t*)^2^. Δ*n*^2^ can be neglected due to Δ*n* ≪ *n*_0_. So the dielectric distribution can be expressed as

. (S2)

Since the RF signal has the same phase velocity with optical modes, the phase-matching condition between *q* and *β* is automatically satisfied. The incident electric field of all order modes is given by

$E(t,z)=\sum_{n} a_{n}(z)e^{i(\omega_{n}t-\beta_{n}z)}$, (S3)

where *a_n_*(*z*) represents the amplitude of *n*th-order mode. *ω_n_* = *ω*_0_ + *n*Ω and *β_n_* = *β*_0_ + *nq* (*n = ±*1, *±*2, …) are the frequency and propagation constants of the *n*th-order mode. The electric field is subject to the time-dependent wave function­­

$\nabla^{2}E(z,t)-\frac{1}{c^{2}}\frac{\partial^{2}}{\partial t^{2}}\left[\varepsilon(z,t)E(z,t)\right]=0$. (S4)

Substituting Eqs. (S2) and (S3) into Eq. (S4), we have

. (S5)

By applying the slowly varying amplitude approximation, Eq. (S5) can be rewritten as

. (S6)

Here *ω_n_*_±1_ = *ω_n_* ± Ω and *β_n_*_±1_ = *β_n_* ± *q*. By substituting (*n* ± 1) by *n*, we have

. (S7)

So the coupled-mode equation for the *n*th-order mode can be rewritten as

, (S8)

where the coupling strength between the adjacent modes is

$\kappa=\frac{n_{0}\Delta n\omega_{n}^{2}}{2\beta_{n}c^{2}}=\frac{\Delta nk_{0}}{2}$. (S9)

We define the form of the mode amplitude as |Ψ(*z*)〉 = ∑­*_n_a_n_*(*z*)|*n*〉. Then the coupled-mode equation can be rewritten as

$i\frac{\partial}{\partial z}|\Psi(z)\rangle=H|\Psi(z)\rangle$. (S10)

The Hamiltonian *H* of the system is given by

. (S11)

The Bloch mode in frequency lattice is an infinite width frequency comb *ψ_n_ = a*_0_exp(*ink_ω_*Ω)exp(*ik_z_z*), with *k_ω_* being the Bloch wavevector and *k_z_* the propagation constant. Solving the coupled-mode equation, the output amplitude of the *n*th-order mode is

, (S13)

*­*where *m_φ_* = 2*κL* is the modulation depth with *L* being the length of PM. *J_n_* denotes the *n*th-order Bessel function. So we obtain the amplitude of the spectrum as |*a_n_*| = *a*_0_*|J_n_*(*m_φ_*)|.

The spectrum evolution can also be analysed from the view of phase modulation. We assume a single-frequency input *E_in_* = *a*_0_exp(*iω*_0_*t*). The phase modulation imposes a time-varying phase factor on optical mode. By applying Jacobi-Anger expansions, the output is given by

. (S14)

By performing the Fourier transform, we obtain the complex amplitude of the *n*th-order frequency mode as

, (S15)

which is the same with Eq. (S13). Thus the spectrum dynamics governed by the coupled-mode equation can also be described from the perspective of phase modulation.

**II. Spectrum evolution for the breathing pattern**

In the fibre-loop circuit, the spectrum evolution for a broad pulse with a width of *T*_0_ = 140 ps is shown in Fig. S1. The modulation depth and frequency are *m_φ_* = 0.5 and Ω = 10 GHz, respectively. For time detuning *τ* = 2 ps, the spectrum evolution exhibits the self-focusing “breathing pattern” with oscillation period *Z*_BO_ = 50 roundtrips, as shown in Fig. S1a. With the increase of the number of roundtrips *l*, the evolution of the *n*th-order frequency mode *a_n_*(*l*) is given by^1^

, (S16)

As shown in Fig. S1b, the simulation results of the amplitude for zero-, first- and second-order frequency mode are respectively denoted by the red, blue and black circles. The simulation data coincide well with the theoretical results plotted by the curves. The periodical change of mode amplitudes verifies the oscillation evolution of the spectrum.


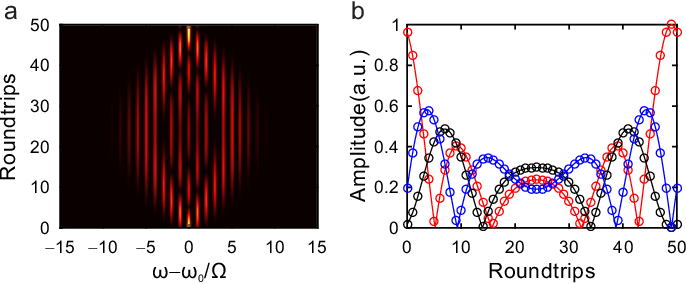


**Fig. S1** **a** Spectrum evolution for pulse with width *T*_0_ = 140 ps. **b** Simulation and theoretical results of the mode amplitudes for zero-, first- and second-order frequency mode.

**III. Effects of non-flat gain and fibre dispersion**

In our work, the spectrum BOs have been realized in an optical fibre-loop circuit. For an ideal situation, the erbium-doped fibre (EDF) compensates for the propagation and insert loss with a flat gain. The fibre dispersion can be compensated by dispersion-compensating fibre (DCF). However, the gain and fibre dispersion cannot be accurately controlled. Here we numerically simulate the spectrum evolution under the effects of non-flat gain and fibre dispersion.

Firstly, we analyse the effect of the non-flat gain. The normalized gain spectrum of the EDF measured in our experiment is shown in Fig. S2a. The centre frequency of the incident pulse is set to 193.93 THz (1555 nm). Then the gain spectrum in the range [*f*_0_ – 400 GHz, *f*_0_ + 400 GHz], marked by the red box in Fig. S2a, is depicted by the red curve in Fig. S2b. The gain curve can be fitted by a Gaussian function *A*_g_ = exp[− (*f* − *δf* )^2^/(2Δ*f* ^2^)] with *δf* = 90 THz and Δ*f* = 550 GHz. The blue curve in Fig. S2b indicates the spectrum envelope of the pulse with width of *T*_0_ = 3.5 ps. As the optical pulse propagates in the fibre-loop circuit, the high-frequency components of the pulse will be attenuated due to the lower gain. The experimental data of the BOs for *T*_0_ = 3.5 ps are shown in Fig. S2c. As the number of roundtrips increases, the spectrum width gradually narrows. The simulation results of spectral evolution with the non-flat gain are shown in Fig. S2d, which is consistent with the experimental data. For a broader pulse with *T*_0_ = 140 ps, the experimental and simulation results are shown in Fig. S2c and S2d, respectively. Due to the variation in the gain profile, the spectrum widths also decrease as the number of roundtrips increases.


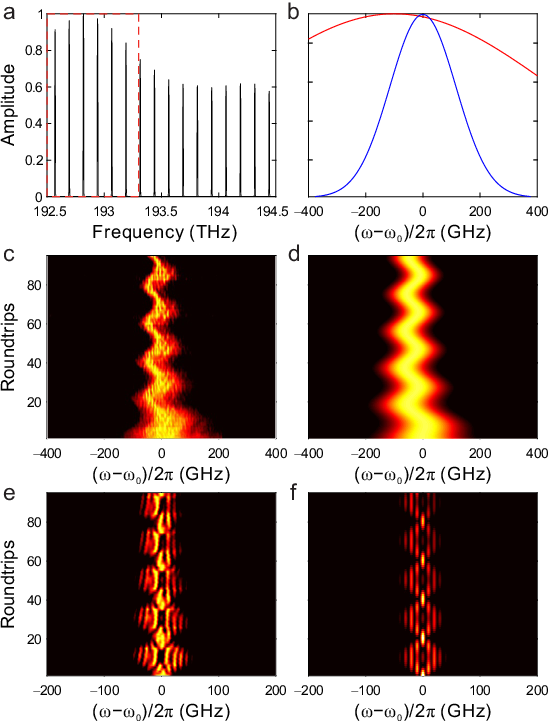


**Fig. S2 a, b** Gain spectrum of the EDF. **c,** Experimental results of the spectrum evolution for pulse width of *T*_0_ = 3.5 ps. **d,** Simulation results corresponds to (**c**). **e, f** Experimental and simulation results of the breathing pattern for *T*_0_ = 140 ps.

Secondly, we simulate the spectrum evolution under the effect of a slight fibre dispersion, as shown in Fig. S3. The spectrum performs a breathing pattern with *T*_0_ = 140 ps. The values of time detuning are set to *τ* = 5 and 8 ps, respectively. As optical pulse propagates in fibre loop, the spectrum is affected by 2.16 ps^2^ group delay dispersion and the non-flat gain mentioned above. The non-flat gain of the EDF results in the asymmetric distribution of the spectral amplitude. As the number of roundtrips increases, the dispersion accumulates and induces remarkable time delays for individual frequency components. Thus the pulse will be modulated by the PM with distinct modulation phases in each roundtrip, which induce the distortion of the spectrum wavepacket.


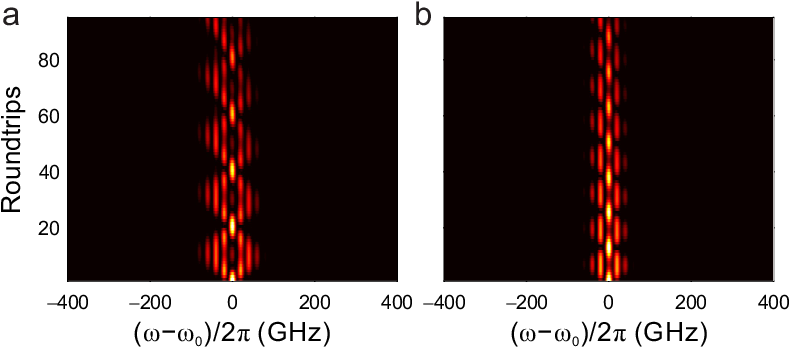


**Fig. S3** Simulation result of the breathing pattern under the effects of fibre dispersion and non-flat gain. The values of time detuning are (**a)** *τ* = 5 ps and (**b)** *τ* = 8 ps, respectively.

**IV. Numerical simulation of pulse spectrum in a fibre-loop circuit**

The spectrum evolution of the pulse circling in the fibre-loop circuit can be numerically simulated by the modified spit-step algorithm. Here we level out the fibre dispersion and loss in the fibre loop. Considering a time detuning *τ* between pulse and modulation signal, the optical pulse in each roundtrip is governed by

, (S17)

where *A_l_* denotes the complex amplitude of the pulse in the *l*-th roundtrip. *m_φ_*, Ω and *φ*_0_ are modulation depth, frequency and initial phase of the RF signal. After each roundtrip, half the energy of the pulse is coupled out from the fibre loop through the 50/50 coupler and enters the long DCF. Under the effect of the dispersion in DCF, the evolution of pulse is governed by modified Schrödinger equation^2^

$i\frac{\partial}{\partial z}A_{l}(z,t)-\frac{\beta_{2}}{2}\frac{\partial^{2}}{\partial t^{2}}A_{l}(z,t)=0$, (S18)

Here *β*_2_ is the group velocity dispersion (GVD) coefficient. The high-order dispersion is neglected here. Thus the temporal waveform of the pulse *ψ_l_* is given by

, (S19)

where *L_D_* is the length of the DCF.

Furthermore, the resolution of DFT technique is given by^3-5^

$\Delta f=\sqrt{\frac{1}{\beta_{2}L\pi}}$, (S20)

which is determined by the fibre dispersion. In our experiment, the parameters are given by *β*_2_ = 210 ps^2^ km^−1^ and *L_­D_* = 16 km. Thus the spectrum resolution is Δ*f* ≈ 9.8 GHz, which basically satisfies the requirement in our experiment.

1. Pertsch, T. *et al*. Optical bloch oscillations in temperature tuned waveguide arrays. *Physical Review Letters* **83**, 4752-4755 (1999).

2. Agrawal, G. P. Nonlinear Fiber Optics. 4th edn. (Boston: Academic Press, 2007).

3. Liu, X. M., Yao, X. K. & Cui, Y. D. Real-time observation of the buildup of soliton molecules. *Physical Review Letters* **121**, 023905 (2018).

4. Goda, K., Tsia, K. K. & Jalali, B. Serial time-encoded amplified imaging for real-time observation of fast dynamic phenomena. *Nature* **458**, 1145-1149 (2009).

5. Goda, K. e*t al*. Theory of amplified dispersive fourier transformation. *Physical Review A* **80**, 043821 (2009).
